# Health Literacy, Health Behaviors, and Body Mass Index Impacts on Quality of Life: Cross-Sectional Study of University Students in Surabaya, Indonesia

**DOI:** 10.3390/ijerph182413132

**Published:** 2021-12-13

**Authors:** Junaidi Budi Prihanto, Endang Sri Wahjuni, Faridha Nurhayati, Ryota Matsuyama, Miwako Tsunematsu, Masayuki Kakehashi

**Affiliations:** 1 Department of Health Informatics, Graduate School of Biomedical and Health Science, Hiroshima University, Hiroshima 734-8553, Japan; rmatsuyama@hiroshima-u.ac.jp (R.M.); tsunematsu@hiroshima-u.ac.jp (M.T.); kakehashi@hiroshima-u.ac.jp (M.K.); 2 Department of Sport Education, Faculty of Sport Science, State University of Surabaya, Surabaya 60213, Indonesia; endangwahjuni@unesa.ac.id (E.S.W.); faridhanurhayati@unesa.ac.id (F.N.)

**Keywords:** quality of life, health behaviors, health literacy, body mass index, university student, cross-sectional study

## Abstract

University student life is an important transformation stage with many potential factors that can impact negatively on the Quality of Life (QOL) and the adoption of unhealthy behaviors. Health literacy (HL), Health Behaviors (HBs), and Body Mass Index (BMI) have been found to be essential components in influencing QOL, in addition to socioeconomic determinants. To identify the influential factors of QOL, we performed a cross-sectional survey in a university, and 955 undergraduate students were enrolled as respondents. We measured two different aspects of HL, i.e., Comprehensive Health Literacy (CHL) and Functional Health Literacy (FHL). Overall, QOL scores in four domains did not differ, regarded as medium with averages ranging from 57.00–63.98, and no significant difference between male and female students. In multivariate analysis, CHL had a significant positive influence on all domains, while FHL only affected the psychological and environmental domains with negative associations. Academic performance had a significant positive association with physical and psychological domains. Students from education majors had higher QOL in the psychological and social domains. Moreover, students with normal or underweight BMI status had better psychological QOL, and physical exercise had a positive association with the social relationship and environmental domains. The findings confirmed that CHL and FHL had significant associations with QOL domains in different ways that should be addressed accordingly. Public health practitioners should carefully empower students to use both CHL and FHL as decision-making skills by incorporating them into related curricula.

## 1. Introduction

University students are a distinct population characterized by the transition process from adolescence to young adulthood, being challenged by academic burdens, economic pressure, expectations of a better future, social identity, independent life far from parents, and uncertainty of life after graduation [1,2,3,4,5]. Burdens during study life at university can affect students’ mental health, sleep pattern, health behavior, and quality of life which can cause a detrimental effect on health or even lead to mortality [6,7,8,9,10,11]. To prevent adverse impacts on physical and psychological health, students’ quality of life and health behaviors should be monitored in order to address problematic issues appropriately.

Quality of Life (QOL) is defined by The World Health Organization (WHO) as “an individual’s perception of their position in life in the context of the culture and value systems in which they live and in relation to their goals, expectations, standards, and concerns” [12]. A short version instrument to measure QOL was developed by the WHO working group WHOQOL-BREF, targeting four domains in QOL; (i) physical health, (ii) psychological health, (iii) social relationships, and (iv) environmental health [13]. This questionnaire was designed for general purposes and has been utilized in the measurement of QOL in research (e.g., medical and social science) and by the general public [14,15,16,17]. Considering the importance of QOL on university students’ lives, understanding the influential factors of QOL will be beneficial for directing effective intervention, and only a limited number of studies have been conducted in this regard in Indonesia.

Health Literacy (HL) is recognized widely as a strong influencing factor for QOL proved by studies in the general public [18,19] and systematic [20,21]. Better HL considered to increase individual autonomy and personal empowerment that could lead to individual overall growth toward a higher QOL [21]. HL is described by WHO as a person’s ability to acquire and interpret health information and services, as well as to use that information to make better health decisions [22]. Functional and comprehensive health literacy are the two categories of HL that are often utilized in scientific research [21,23]. The ability to read and comprehend health-related information is referred to as Functional Health Literacy (FHL) [23], whereas Comprehensive Health Literacy (CHL) is the ability to find, interpret, evaluate, and use the information to make healthy decisions [21].

Health Behaviors (HBs) are defined as all individual activities that are taken intentionally or unintentionally and affect their health. HBs could become beneficial to health (e.g., such as diet, physical training, healthy sleeping pattern) or become a health risk factor (e.g., smoking, alcohol drinking, substance use) [24]. Individuals with HBs that can cause risk to health such as smoking, drinking alcohol, and substance abuse proven to have lower QOL [25,26,27,28]. While people practicing the HBs with health benefits (e.g., physically active, enough sleeping hours) have better QOL [17,29,30,31].

BMI as a health outcome that demonstrate the individual nutritional status had been proved as a predictor of mortality, morbidity, and health expectancy [32,33]. Preceding studies also showed that BMI influence QOL; people with overweight and obesity had been reported to have lower QOL [34,35]. QOL from people with the obese condition showed the worst status by stigma received from the community that they belonged [36]. A lower level of HL was also found in the people with obesity compared to those who had normal BMI [37,38].

Socioeconomic factors have been recognized as influential factors for QOL in many cultural settings. The QOL physical health domain has been significantly associated with gender, economic class, years/level of study, type of residence, employment status level, family structure, and father’s occupation, while gender, age, year/level of study, academic load, type of residence, marital status, education level, and family structure had significant correlation with the psychological domain [29,39,40,41]. The QOL social relationship domain was correlated with age, economic class, year of study, marital status, education level, and family structure [29,39,40,41]. Gender, economic class, years of study, monthly allowance, type of residence, marital status, family structure, and father’s occupation were associated with environmental health [29,39,40,41]. Major/study field of university student also influences the QOL, preceding studies only focus on the comparison of different specialty in medical students [30], medical with other health profession students [42], or medical with non-medical [43,44], although characteristics of educational major curricula have potentially influence student QOL.

**Figure 1:**
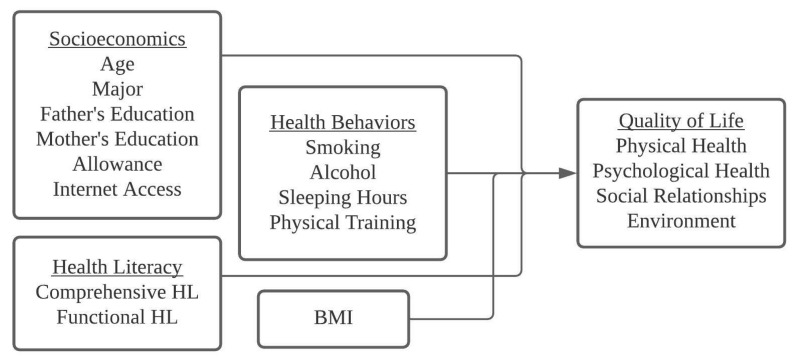
Hypothetical association between socioeconomic determinants, HL, HBs, BMI, and QOL.

This research was guided by two questions: (i) What is the effect of health literacy on the quality of life among Indonesian undergraduate university students when socioeconomic considerations are taken into account? (ii) What is the impact of health behaviors on quality of life? We measured QOL, HL, HBs, and BMI in university students from various faculties and majors in Surabaya, Indonesia to answer these questions. Then, taking the influence of relevant socioeconomic determinants into account, we investigated the cross-sectional relationship between QOL, HL, HBs, and BMI. The hypothetical associations between socioeconomic, health literacy, health behaviors, and quality of life are shown in Figure 1.

## 2. Materials and Methods

### 2.1. Description of Study Design, Population, and Sample

This research used a cross-sectional design and conducted on undergraduate students of a university in Surabaya, Indonesia, from December 2019 until mid-January 2020. Ethical approval obtained for this research was issued by Hiroshima University (7 August 2019; number E-1705) and by Surabaya State University Research and Community Service Bureau (B/72445/UN38.9/LT/02/2019).

We used an anonymous self-administered questionnaire, and respondents participated voluntarily after informed consent. The respondents were able to ask questions or cancel participation during data collection. Two investigators from the research team explained the study purpose, how to participate, how to answer the questions, and guided the respondents during the data retrieval process. We provided a ballpoint pen, snack, and drink to respondents in appreciation for their participation.

Surabaya State University as a study site was one of four public universities in Surabaya, had seven faculties, one graduate school, 112 courses, 1027 lecturers, and 16,466 students [45,46]. A convenient sampling method was used to recruit participants from seven faculties (Engineering; Economics; Mathematics and Natural Sciences; Social Sciences and Law; Sports Science; Languages and Arts; and Education) with a total of 61 undergraduate courses [45,46]. First, we sampled four undergraduate courses from each faculty balancing the proportion of course types selected as the study site. Courses were classified into two categories based on the presence of pedagogical and educational material in the curriculum: education (teacher training and other jobs in the education field) and non-education. For faculties that have less than four undergraduate courses, all courses were selected. Lastly, we chose one class of 30–40 students within each course. Inclusion criteria for respondents were all the student who willing to participated voluntarily in the class that was selected, already studied minimum one semester, and in good health condition.

The sample size was calculated using the G*Power version 3.1.9.2 (Faul et al., Kiel University, Kiel, Germany) for linear multiple regression, two-tail analysis, α error probability 0.05, power (1 − β error probability) 0.80 and the result was 409 samples. With the consideration of the different characteristics of undergraduate students from 7 faculty, 27 courses selected from education or non-education majors, and a maximum number of 40 undergraduate students in each class, we prepared 1080 questionnaires. A total of 955 students participated in this study, 25 respondents’ data (2.62%) were excluded from the analysis because they answer less than 14 questions or have missing answers more than two for CHL (HLS-EU-Q16).

### 2.2. Measures

#### 2.2.1. Quality of Life

The WHOQOL-BREF was used to measure the quality of life as the result of an individual’s perspective on their place in life in relation to their objectives, expectations, standards, and concerns in the context of the culture and value systems in which they live [12]. There were four domains in this instrument: i.e., physical health, psychological health, social relationships, and environmental health. The four domains used in this study were measured by a total of 26 questions and transformed into 100 scales using the scoring method following the WHOQOL-BREF working group instruction manual [13]. The higher the value is the higher QOL in each domain. The instrument reliability measured by the WHOQOL Group using Cronbach’s α produce values of 0.80, 0.76, 0.66, and 0.80 for physical, psychological, social relationships, and environment domains respectively. Construct validity measured by domain and overall QOL correlation produce significant positive r_s_ for all domains (physical health: 0.65, psychological health: 0.67, social relations: 0.44 and environment: 0.57) [47]. Current study Cronbach’s α for WHOQOL physical, psychological, social relationship and environment were 0.535, 0.521, 0.493 and 0.741 consecutively. We found that reliability analysis in the data show different result by faculty with Cronbach’s α for physical 0.616–0.463, psychological 0.576–0.454, social relationship 0.678–0.297, and environment 0.829–0.597.

#### 2.2.2. Comprehensive Health Literacy (CHL)

The HLS-EU-16 that consists of 16 questions is a condensed version of the HLS-EU-47, which was created to assess people’s ability to seek, comprehend, judge, and apply health information to maintain and improve their health at a community level [48]. Permission to use HLS-EU-Q16 was obtained by email from the coordinator of the European Health Literacy Project [49]. This self-reported instrument used four-degree responses of “very easy”, “easy”, “difficult”, and “very difficult” options (Likert-type) and one option “don’t know”. Responses from 16 questions were transformed into binary values for HLS-EU-16 grading, coded 1 for “very easy” and “easy,” and 0 for “difficult” and “very difficult”. Respondents who chose “don’t know” responses or decline to answer counted as missing, and we only analyzed data from respondents who answered a minimum of 14 questions or only had a maximum of two missing values. All answers were summed as the total score of CHL ranging from 0–16 and converted into three categories, “sufficient” (>12 scores), “problematic” (9–12 scores), and “inadequate” (<9 scores), classifications proposed by Pelikan et al. [48]. HLS-EU-Q16 reliability and validity had been proved to be consistent and adequate as reported in Spain (Cronbach’s α (α): 0.982, Inter-Class Correlation (ICC): 0.923) [50], India (α: 0.95, Tucker–Lewis Index (TLI) and Goodness of Fit Index (GFI) > 0.8) [51], France (α: 0.81, Kappa: 0.36) [52], and Iceland (α: 0.88, Corrected Item—Total Correlation (CITC) > 0.40) [53]. In our study, the Cronbach’s α for overall HLS-EU-Q16 was 0.793.

#### 2.2.3. Functional Health Literacy (FHL)

The Newest Vital Sign (NVS) is a tool that evaluates people’s capacity to absorb and comprehend health-related information. People’s capacity to utilize health knowledge to read and understand the information in words and numbers was assessed using ice cream nutrition labels and six-question items. The total number of correct answers was used as the score of FHL in the study, with four or more correct answers indicating average literacy, two to three correct responses showing marginal literacy, and fewer than two correct answers indicating low literacy [54]. NVS had consistency and validity as showed by preceding studies in America (Cronbach’s α (α): 0.76, criterion validity r = 0.59, *p* < 0.001) [54], in Netherland (α: 0.76, construct validity Pearson r = 0.53 − 0.20) [55], in Japan (α: 0.72, criterion validity Pearson r = 0.72 − (−0.30)) [56], in Italia (α: 0.74, construct validity Pearson r = 0.58) [57], and in Taiwan (α: 0.70, construct validity Pearson r = 0.58 − 0.18) [58]. The reliability of NVS in our study was α: 0.567, *p* ≤ 0.001.

#### 2.2.4. Health Behaviors

Health Behaviors (HBs) were measured by the International Health Behaviors Surveys (IHBS) Questionnaire [59]. The IHBS Questionnaire was developed to measure health-related behavior, risk awareness, and associated attitudes of university students worldwide using standardized measurement allowing for direct comparisons between young adults from different nations and cultures. This questionnaire has high and proven validity and reliability. It is translated into 18 languages and had been used in 24 countries [59,60]. Every response was transformed to a categorical scale; for binomial variables, we coded 1 if the response indicated a benefit to health and 0 if it indicated a health risk, whereas the variables with more than two categories were given descending or ascending ordinal numbers according to the benefit of health.

Smoking was measured by selecting one out of eight statements in the smoking habits section. The statements “(a) I have never smoked, even tried smoking a cigarette” and “(b) I’ve only tried one or two cigarettes” were coded as 1, and other statements that were ordinal responses about the degree of smoking were coded as 0. The alcohol variable was derived from four statements in the alcohol part, the statement “(a) Not a drinker of alcohol” was coded 1 and all the other gradual magnitude statements of alcohol consumption were coded 0. The sleeping variable was converted from the average hours of sleeping in 24 h reported by respondents. It was coded 1 for teens (13–17 years) if 8–10 h and for adults (18–60 years) if 7 or more hours, while any other than these recommended values was coded 0 [61,62]. Physical exercise was taken from the question “Over the past 2 weeks (14 days), have you taken any exercise, (e.g., sport, physically active pastime)?” the statement “Yes” was coded 1, and “No” was coded 0.

#### 2.2.5. Body Mass Index (BMI)

BMI was additionally collected as a health outcome for evaluating the nutrition status among respondents. The BMI status of the respondents was computed using the BMI formula from their self-reported weight and height data, and they were categorized using the WHO BMI score based on their age [63,64]. BMI status was coded as 1: underweight, 2: normal, or 3: overweight/obese.

#### 2.2.6. Socioeconomic Determinants

Socioeconomic determinants collected in this study were gender, age, academic performance, father’s education, mother’s education, allowance, and internet access, which were all self-reported. Allowance was considered as monthly pocket money that respondents usually have and use as the measure for economic status. The variable included three categories: high (>USD 35), medium (USD 14–35), and low (<USD 14).

### 2.3. Statistical Analysis

To describe the distribution of data, categorical variables were summarized by proportions, while continuous variables were assessed using mean and standard deviation. The chi-square test was used for finding associations between Gender and other categorical variables, in addition, the Mann–Whitney test and Kruskal–Wallis test were used for analyzing the associations between the four domains of quality of life and other variables. The Generalized Linear Model (GLM) with the gamma log-link distribution was used to evaluate the association between four QOL domains and socioeconomic determinants, CHL, FHL, HBs, and BMI by setting each QOL domain as a response variable and other variables as explanatory variables. The GLM gamma distribution modeling was chosen because the distribution of all QOL domains as response variables in linear regression was not multivariate normal distribution. We used two different models, one involving CHL and the other FHL, to evaluate whether the two different HLs will produce different influences on QOL. The positive or negative influence of predictor variables will be evaluated according to the exponential β (exp β) coefficient whether it was larger than 1 or not, where β is a partial regression coefficient. The same procedure was applied to each value of categorical variables, with a selection of one value as reference (i.e., β = 0 or exp β = 1). IBM SPSS Statistics for Windows version 25 (IBM, Armonk, NY, USA) was used to conduct statistical analyses. The significance level (alpha) was set at 0.05 in each statistical analysis.

## 3. Results

### 3.1. Respondent Characteristics

The data were collected from a total of 27 undergraduate courses from seven faculties of a university. A total of 955 students participated in this study. Using the inclusion and exclusion criteria from CHL (HLS-EU-Q16), 25 respondents (2.62%) needed to be excluded. The data of 930 respondents were analyzed; among those, 625 (67.20%) were female. Table 1 describes the characteristics of the study participants.

There was no significant difference in age distribution between female and male participants, with a mean age of 19.81 years old. Most participants had a high academic performance with a GPA mean of 3.41 (on a scale of 4), and female students had better academic performance (Mean 3.43, *p*-value = 0.000). There was the same trend in father’s and mother’s educational background: Junior and Senior high school level had the largest proportion (59.03% of fathers, 58.28% of mothers); while university and postgraduate constituted the second-largest proportion (29.25% of fathers, 28.60% of mothers). In general, there were no differences in father’s and mother’s education level though male participants had slightly better father’s and mother’s educational levels compared with female students.

Students’ monthly allowance showed the majority of students (50.32%) belonged to high economic status (>USD 35) and female students had better economic status compared to male students (*p*-value = 0.012). Most participants had access to private internet, but 26.88% did not, and female participants had a slightly larger internet volume subscription (*p*-value = 0.000).

Different results in health literacy characteristics were found in CHL and FHL measurements. In CHL, most of the respondents had sufficient CHL (68.28%) while only 17.63% had the same level of FHL (i.e., average). A difference in health literacy by gender was found in FHL (*p*-value = 0.001), where female participants (19.52%) had better FHL than male participants (13.77%).

There was significant difference (*p*-value < 0.001) in BMI between male and female students. Male students had larger proportion of normal (60.66%) and Obese (24.59%) BMI categories compare to female student (normal = 57.28%, obese = 17.28%). Only in the underweight category did female students (25.44%) show a larger proportion than male students (14.75%).

For health behavior, female students had better behavior on smoking (*p*-value = 0.000) and alcohol consumption (*p*-value = 0.000), whereas male students had better behavior in physical exercise (*p*-value = 0.000) In contrast, there was no difference in sleeping hours between the genders. Overall, the prevalence of negative health behaviors such as smoking and drinking alcohol were low at 5.5% and 7.7% respectively. However, the proportions of physical exercise and sleeping hours were quite concerning because neither gender got enough sleep (54.20%) nor engaged in adequate exercise (41.30%).

### 3.2. QOL Association with Socioeconomic Determinants, CHL, FHL, HBs, and BMI

Associations between four domains of QOL with socioeconomic determinants, CHL, FHL, and HBs are shown in Table 2. For socioeconomic variables, academic performance (all *p*-value < 0.05) were associated significantly with four domains while major was associated with three domains: psychological (*p*-value < 0.01), social relationship (*p*-value < 0.05) and environmental health (*p*-value < 0.01). Age was associated with physical (*p*-value *=* 0.016) and environmental health (*p*-value = 0.003), and father’s educational background (*p*-value < 0.01), allowance (*p*-value < 0.001), and internet access (*p*-value *<* 0.01) were associated with environmental health. CHL had a significant association with all four domains (all *p*-value < 0.001), whereas FHL only had an association with physical (*p*-value < 0.001) and social relationships (*p*-value < 0.05). BMI as a health outcome had a significant association with psychological health (*p*-value < 0.001) and social relationships (*p*-value *<* 0.05). Not many HBs had an association with QOL, the ones that had associations were sleeping hours to physical health (*p*-value < 0.05) and physical exercise to social relationships (*p*-value < 0.001) and environmental health (*p*-value < 0.01).

### 3.3. Multivariate Generalized Linear Models of the Four Domains of QOL with Socioeconomic Determinants, HBs, BMI, and CHL

We performed multivariate analysis using Gamma log link GLMs for the four QOL domains using socioeconomics, HL, BMI and HBs as predictor variables. We distinct CHL and FHL in different model because they both measured HL in different approach and could cause multicollinearity if used together in same model. The results of GLM gamma modeling using CHL are shown in Table 3. For the physical health domain model, only academic performance (exp β: 1.062, *p*-value < 0.05) and CHL (inadequate: 0.878, *p*-value < 0.001; problematic: 0.878, *p*-value < 0.001) had significant positive influences. Academic performance (1.080, *p*-value < 0.01), CHL (inadequate: 0.900, *p*-value < 0.01; problematic: 0.950, *p*-value < 0.001), and BMI (underweight: 1.065, *p*-value < 0.01; normal: 1.071, *p*-value < 0.001) had a significant positive influence on the psychological health domain. Major (non-education: 0.959, *p*-value < 0.05), CHL (inadequate: 0.874, *p*-value < 0.01; problematic: 0.934, *p*-value < 0.01), and physical exercise (no: 0.940, *p*-value < 0.01) variables positively influenced the social relationships domain. Environmental health was influenced by CHL (inadequate: 0.854, *p*-value < 0.001; problematic: 0.937, *p*-value < 0.001) and physical exercise (no: 0.959, *p*-value < 0.01).

### 3.4. Multivariate Generalized Linear Models of the Four Domains of QOL with Socioeconomic Determinants, HBs, BMI, and FHL

Gamma GLMs for the four QOL domains using FHL as the health literacy indicator instead of CHL are shown in Table 4. For the QOL physical health domain, academic performance (exp β: 1.078, *p*-value < 0.05), father’s education (elementary and below: 0.950, *p*-value < 0.05), and FHL (limited: 1.048, *p*-value < 0.05) had significant influences. Academic performance (1.088, *p*-value < 0.01), major (non-education: 0.962, *p*-value < 0.001), and BMI (underweight: 1.065, *p*-value < 0.01; normal: 1.074, *p*-value < 0.001) had a significant influence on the QOL psychological health domain. Major (non-education: 0.956, *p*-value < 0.05), FHL (limited: 1.075, *p*-value < 0.01), BMI (normal: 1.054, *p*-value < 0.05) and physical exercise (no: 0.948, *p*-value < 0.01) variables had significant influence on the QOL social relationships domain. QOL Environmental health had positive significant association with academic performance (1.076, *p*-value < 0.05), father’s education (elementary and below: 0.938, *p*-value < 0.01), mother’s education (junior and senior high: 0.970, *p*-value < 0.05), allowance (medium: 0.952, *p*-value < 0.001), internet access (< 10 giga: 0.968, *p*-value < 0.05) and physical exercise (no: 0.964, *p*-value < 0.01).

## 4. Discussion

This study focused on the measurement of Quality of Life (QOL) and its association with socioeconomic determinants, Health Literacy (HL), BMI, and Health Behaviors (HBs) among Indonesian undergraduate students. We used two types of health literacy variables and analyzed them in two different multivariate models. Health literacy proved to be a prominent independent variable (IV) that affects the quality of life. Whereas Comprehensive Health Literacy (CHL) had a positive influence for all of the QOL domains, Functional Health Literacy (FHL) only affected physical health and social relationships with a negative association. Academic performance was the most influential socioeconomic IV in both multivariate models, significantly and positively affecting all QOL domains’ social relationships. Education major students scored higher in psychological health and social relationships QOL, while students with higher allowance had higher QOL in environmental health with both multivariate models. Normal BMI status was associated with higher QOL in the psychological and social relationship domains. As for the health behavior variables, only physical exercise had a positive association with the social relationship and environmental health domains.

For a better understanding of health literacy’s effect on QOL, we utilized two types of HL measurement, HLS-EU-Q16 for CHL and NVS for FHL. Both HL instruments have been proved to be valid and reliable and are also widely used in the preceding literature which makes comparison with this study easier. For CHL that measured students’ perception in searching, comprehending, assessing, and implementing health information in a health setting, the results were quite good with 635 (68.28%) students reaching the sufficient category. In contrast, for FHL, which measures the ability to read, understand and calculate simple math in health information, a concerning outcome was obtained: only 164 (17.63%) students scored in the average category. The results on CHL and FHL in the undergraduate university students in Surabaya had a similar trend with the survey research we conducted on 1066 students in 15 high schools in Surabaya [65]. These commonly observed results of disparity between the CHL and FHL suggest the existence of a problem in health education and promotion at educational institutions that need to be addressed properly to improve the health outcomes and wellbeing of the students. This is because, when there is a gap between CHL and FHL, it means that there were individuals that over-estimated their capability in health literacy and had low-level ability in word and math literacy in health settings.

Based on the preceding study and meta-analysis, HL is the individual capacity that influences self-efficacy and decision-making ability that is needed to achieve better health outcomes including QOL [21,66]. In this study, we validated the difference in the contributions of CHL and FHL to QOL. Firstly, CHL was positively associated with all four domains of QOL. These were similar to the results from the preceding study that used the same type of health literacy instrument conducted by Panagioti et al. in the UK general practice cohort study [67]; another study on students in Indonesia reported the association on three domains except physical health [68]. Secondly, FHL had a negative association with the physical health and social relationship domains, a different result from the Veiga and Serrao study of elderly Portuguese [69], which reported a positive weak association among QOL domains even though showing quite a similar distribution of FHL levels as this study. A different result of CHL and FHL could be understood because CHL and QOL instruments used in this current study were both measured by perception while FHL was measuring the literacy and numeracy ability in a health context. Moreover, when the investigator asked a follow-up question about whether respondents always read the nutrition fact when buying food products in the market, most of them only checked expired date without reading the ingredients and nutritional value. Based on the result of the FHL negative associations with QOL domains in this study, we suspect that this phenomenon could be caused because the respondents came from more homogenous population (university student in same age group and relatively same academic ability) compare with Veiga and Serrao study population (Elder population with more heterogenous characteristic). Study with bigger and heterogenous population is needed to investigate whether this phenomenon is caused mainly by variability in respondent characteristics or by cultural differences.

Academic performance was found to be the most influential socioeconomic variable on QOL domains in both CHL and FHL models with positive associations with the physical, psychological, and environmental health domains. The cognitive ability that strongly influences academic performance could be the reason behind the result [70,71]. Preceding studies themselves also shown inconsistent results. The same result with our study was shown by a study in Trinidad and Tobago [42], and a positive association with all QOL domains was displayed in Alfaisal University (Saudi Arabia) [72]. In contrast, negative associations were shown with psychological health and social relationship QOL domains in the study of medical students in King Abdul Azis University (Saudi Arabia) [73]. The different results in QOL by Academic performance could be related to the study load, educational environment, expectations, and cultural values. Major in this study showed that education students had better psychological health and social relationships compared with non-educational course students. This could be because the education course curriculum contains psychology and pedagogical subjects that may affect QOL. Previous studies also showed such influences of different majors on associations with the QOL domains [42,43].

Some socioeconomic determinants demonstrated significant associations with the four QOL domains. In particular, allowance as the economic measurement in this study showed a positive association with environmental health, similar to a preceding study in a Brazilian university that showed positive influence on physical, psychological, and environmental health [74]. In contrast, there was a different result in the study of Filipino nursing students, showing a negative association with the environmental domain [29]. In addition, economic variables in the general population showed a positive significant influence on all QOL domains in Pakistan [40], Brazil [39], and France [41]. Other variables in this study, fathers’ and mothers’ educational background only had a positive association with environmental health while preceding studies in the general population showed different results. A QOL study of immigrants in France [41] showed that a better father’s educational background was associated with better in all QOL domains, while the study in Pakistan [40] exhibited influence on all QOL domains except environmental health. Furthermore, internet access in this study showed a negative association with environmental health, while a preceding study in Taiwan [15] showed a positive association with psychological health. The influences on QOL from socioeconomic determinants seemed unstable, suggesting dependence on some unknown variables.

The associations between socioeconomic variables and QOL showed further unique features of the present study’s results. No influence from gender nor age on QOL was one of the most surprising results of this study. This is because most of the preceding studies reported gender differences in QOL, with only one study in Saudi Arabia having the same result [73] as this study. In most studies, males had better physical health [29,30,40,72,74,75,76,77], and psychological health [29,30,40,72,74,77], while females had better social relationships [30,77]. No influence of gender in this study indicated that both genders culturally have the same equality on the social role, expectation, and burden that reflect in the same QOL result. As for age, in a preceding study, positive associations were shown by age variable with physical and environmental health [42], in contrast with another preceding study age exhibiting a negative association with psychological health and social relationships [39]. The possible reason behind no association of age to QOL was the respondents of the current study belong in the same age group (young adult) with the characteristic of better impulse control, sensitivity to possible consequences of conduct, less affected with the behavior-related rewards, and take longer to examine tough situation [78].

In the current study, BMI has a significant negative association with psychological and social relationship domains of QOL, i.e., obese respondents scored lower compared with normal weight. The negative association with the social domain was also reported by a study in Taiwan (2008) [34], Iran (2012) [79], and Brazil (2017) [35]. This could happen because an individual with obesity always struggling with mental distress caused by mood, self-esteem, and body images issues that lower their QOL [36,80]. Only one study in Taiwan (2008) [34] showed the same negative association with social relationships. The reason why a negative association with social relationships was observed because obese individuals experience stigma in form of negative attitudes and discriminatory behavior toward them from the community members that have anti-obese cultural value [36]. Other studies [34,35,79,81,82] also reported a negative association with the physical domain, which is different from our study. This discrepancy could be explained by the difference in acceptance of obese respondents’ physical condition in different population.

Regarding the associations between the health behavior variables and the four domains of QOL, we observed both similarities and differences compared with the results of the present study. In the current study smoking and alcohol had no significant association with any of the QOL domains, this could be caused by the very low smoking (5.9%) and alcohol consumption (7.7%) among the respondents and the restriction of alcohol selling in Indonesia. Only one preceding study reported no association between alcohol and all domains [83], but all the other preceding studies showed a negative association of smoking and alcohol with QOL [25,26,27,28]. There was no association between hours of sleep and QOL in the current study but preceding studies showed positive significant associations with physical and psychological QOL domains [29]. Physical exercise only positively influenced social relationships and environmental health, but previous studies showed it had positive associations with all QOL domains [17,30,31]. The association of QOL with other factors often showed inconsistent direction among different studies. The situation can be understood as the involvement of many factors, none of which is dominant over the others. Further detailed research will be required to clarify this complex relationship.

There are various limitations in this study that should be considered in future research. First, while the sample size was sufficient to meet the study’s objectives, it was deemed insufficient to represent the vast range of Indonesian population characteristics such as ethnicity, as well as the region’s socio-economic and developmental growth. Second, the low Cronbach’s α result from WHOQOL-BREF (physical, psychological, and social relationship domain) and NVS that indicated low reliability should be addressed carefully. We believe this could have happened because Cronbach’s α was influenced by the low covariance of the component variables in rather homogenous population. Third, QOL, CHL, FHL, and HBs were all assessed using self-reporting questionnaires, which could lead to some respondents giving responses that are more socially acceptable than their real status. Furthermore, self-reporting surveys may have led to respondents’ incorrect understanding of the question. We used anonymity and guaranteed privacy of the data in the informed permission statement that was read and explained by investigators before respondents self-filled the questionnaire to promote honesty and diminish the propensity of socially acceptable answers. Investigators guided and answered questions from the respondents as university undergraduate students filled out the questionnaire to reduce inaccurate interpretation of items in the instrument. Finally, due to the cross-sectional character of our research, we were unable to determine if there was a causal relationship between health behavior and health literacy, health-promoting school activities, or other socioeconomic factors. A longitudinal study could help resolve this problem.

## 5. Conclusions

This study confirmed the importance of Health Literacy (HL) for realizing higher QOL. CHL is especially important because it had significant positive relationships with all QOL domains. An increase in CHL would lead to higher QOL. In contrast, results shown for FHL were that its influence was restricted to within physical health and social relationship domains in a negative direction. This meant better reading and understanding ability in the health setting does not always result in better QOL. Overall, socioeconomic determinants showed a positive impact on QOL in both multivariate models, which meant better academic performance, studies in an education major, higher allowance, and better father’s and mother’s educational background would result in higher QOL. BMI as a health outcome had impact on some domains of QOL, with obese respondents have lower psychological health and social relationship domains, but the influence of health behavior variables on QOL was rather restricted: only physical exercise had positive associations with social relationships and environmental health. Overall, students in the education course with better HL, healthier HB, good academic performance, who were physically active, and with non-obese/overweight BMIs had better QOL. The results of this study can be used as a baseline in understanding the importance of HL on QOL in university student life, which has many challenging burdens that can affect health and wellbeing status. Based on our results, appropriate teaching of health literacy as the ability to understand health information and decision-making skills is suggested as a section in a related course in order to realize higher QOL in university life and the future.

## Figures and Tables

**Table 1:** Descriptive statistics of the respondents in the study by gender.

Variables	Female	Male	Total	Stat. Value	*p*-Value
**Socioeconomics**					
Age (Mean, Sd)	19.78 (0.920)	19.87 (0.945)	19.81 (0.929)	−1.189	0.235 ^1^
Academic Performance (Mean, Sd)	3.43 (0.216)	3.38 (0.220)	3.41 (0.219)	−3.890	0.000 ^1^
Major				7.056	0.008 ^2^
Non-Education	264 (42.24%)	157 (51.48%)	421 (45.27%)		
Education	361 (57.76%)	148 (48.52%)	509 (54.73%)		
Father’s Education				24.836	0.000 ^2^
Elementary and Below	83 (13.28%)	26 (8.52%)	109 (11.72%)		
Junior and Senior High	391 (62.56%)	158 (51.80%)	549 (59.03%)		
University and Postgraduate	151 (24.16%)	121 (39.67%)	272 (29.25%)		
Mother’s Education				8.02	0.018 ^2^
Elementary and Below	82 (13.12%)	40 (13.11%)	122 (13.12%)		
Junior and Senior High	382 (61.12%)	160 (52.46%)	542 (58.28%)		
University and Postgraduate	161 (25.76%)	105 (34.43%)	266 (28.60%)		
Allowance				8.927	0.012 ^2^
Low	69 (11.04%)	54 (17.70%)	123 (13.23%)		
Medium	227 (36.32%)	112 (36.72%)	339 (36.45%)		
High	329 (52.64%)	139 (45.57%)	468 (50.32%)		
Internet Access				5.555	0.135 ^2^
No Private Internet	147 (23.52%)	56 (18.36%)	203 (21.83%)		
<10 giga	164 (26.24%)	72 (23.61%)	236 (25.38%)		
>10 giga, <Unlimited	149 (23.84%)	87 (28.52%)	236 (25.38%)		
Unlimited	329 (52.64%)	139 (45.57%)	255 (27.42%)		
**Health Literacy**					
CHL				0.217	0.897 ^2^
Inadequate	33 (5.28%)	14 (4.59%)	47 (5.05%)		
Problematic	167 (26.72%)	81 (26.56%)	248 (26.67%)		
Sufficient	425 (68.00%)	210 (68.85%)	635 (68.28%)		
FHL				13.129	0.001 ^2^
Limited	255 (40.80%)	162 (53.11%)	417 (44.84%)		
Marginal	248 (39.68%)	101 (33.11%)	349 (37.53%)		
Average	122 (19.52%)	42 (13.77%)	164 (17.63%)		
**Health Behaviors**					
Smoking				78.714	0.000 ^2^
No	618 (98.88%)	257 (84.26%)	875 (94.10%)		
Yes	7 (1.12%)	48 (15.74%)	55 (5.90%)		
Alcohol				18.342	0.000 ^2^
No	593 (94.88%)	265 (86.89%)	858 (92.30%)		
Yes	32 (5.12%)	40 (13.11%)	72 (7.70%)		
Sleeping Hour				0.213	0.649 ^2^
Less	348 (55.68%)	165 (54.10%)	504 (54.20%)		
Enough	277 (44.32%)	140 (45.90%)	426 (45.80%)		
Physical exercise				28.963	0.000 ^2^
No	296 (47.36%)	88 (28.85%)	384 (41.30%)		
Yes	329 (52.64%)	217 (71.15%)	546 (58.70%)		
**Health Outcome**					
BMI				16.637	0.000 ^2^
Underweight	159 (25.44%)	45 (14.75%)	204 (21.90%)		
Normal Weight	358 (57.28%)	185 (60.66%)	543 (58.40%)		
Obese	108 (17.28%)	75 (24.59%)	183 (19.70%)		
**Quality of Life**					
Physical Health (Mean, Sd)	57.02 (10.86)	56.97 (11.31)	57.00 (11.01)	−0.407	0.684 ^1^
Psychological Health (Mean, Sd)	62.56 (10.91)	62.37 (13.06)	62.50 (11.65)	−0.180	0.857 ^1^
Social Relationships (Mean, Sd)	61.80 (16.82)	62.99 (16.86)	62.19 (16.83)	−0.654	0.513 ^1^
Environmental Health (Mean, Sd)	64.11 (11.89)	63.71 (12.93)	63.98 (12.23)	−0.218	0.828 ^1^

^1^ Mann–Whitney U, ^2^ chi-square; statistics: Mann–Whitney-U: Z, chi-square: χ^2^.

**Table 2:** Associations between quality of life to socioeconomic determinants, HL, HBs, and BMI.

Variables	Physical Health		Psychological Health		Social Relationship		Environmental Health	
	Mean (Sd)	*p*-Value (Statistics)	Mean (Sd)	*p*-Value (Statistics)	Mean (Sd)	*p*-Value (Statistics)	Mean (Sd)	*p*-Value (Statistics)
**Socioeconomics**								
Age	-	0.016 ^1^ (0.079)	-	0.277 ^1^ (0.036)	-	0.128 ^1^ (0.050)	-	0.003 ^1^ (0.096)
Gender		0.684 ^2^ (−0.407)		0.857 ^2^ (−0.180)		0.513 ^2^ (−0.654)		0.828 ^2^ (−0.218)
Female	57.02 (10.86)		62.56 (10.91)		61.80 (16.82)		64.11 (11.89)	
Male	56.97 (11.31)		62.37 (13.06)		62.99 (16.86)		63.71 (12.93)	
Academic Performance	-	0.009 ^1^ (0.086)	-	0.001 ^1^ (0.108)	-	0.040 ^1^ (0.067)	-	0.005 ^1^ (0.092)
Major		0.099 ^2^ (−1.648)		0.002 ^2^ (−3.101)		0.028 ^2^ (−2.203)		0.002 ^2^ (−3.050)
Non-Education	56.26 (11.70)		60.94 (12.09)		60.24 (16.59) 2		63.07 (12.85)	
Education	55.14 (19.00)		59.78 (25.00)		58.65 (19.00)		61.84 (25.00)	
Father’s Education		0.159 ^3^ (3.683)		0.490 ^3^ (1.426)		0.679 ^3^ (0.774)		0.002 ^3^ (12.663)
Elementary and Below	55.65 (10.98)		62.62 (9.94)		60.68 (17.34)		61.19 (12.47)	
Junior and Senior High	56.77 (10.87)		62.14 (11.96)		62.17 (16.75)		63.72 (11.80)	
University and Postgraduate	58.02 (11.24)		63.16 (11.66)		62.84 (16.81)		65.62 (12.79)	
Mother’s Education		0.824 ^3^ (0.386)		0.644 ^3^ (0.881)		0.983 ^3^ (0.034)		0.071 ^3^ (5.285)
Elementary and Below	56.43 (12.17)		61.71 (11.59)		62.69 (17.82)		62.96 (13.40)	
Junior and Senior High	57.04 (10.57)		62.41 (11.56)		62.01 (16.56)		63.41 (11.82)	
University and Postgraduate	57.18 (11.35)		63.04 (11.89)		62.32 (16.97)		65.61 (12.40)	
Allowance		0.517 ^3^ (1.318)		0.056 ^3^ (5.750)		0.290 ^3^ (2.478)		0.000 ^3^ (18.204)
Low	56.06 (11.51)		60.71 (12.48)		60.39 (19.33)		62.98 (13.89)	
Medium	56.60 (10.70)		62.07 (11.10)		61.72 (16.47)		62.23 (11.21)	
High	57.54 (11.08)		63.27 (11.78)		63.00 (16.37)		65.51 (12.31)	
Internet Access		0.247 ^3^ (4.141)		0.367 ^3^ (3.163)		0.144 ^3^ (5.415)		0.006 ^3^ (12.446)
No Private Internet	57.76 (11.50)		63.04 (12.14)		62.29 (19.48)		65.79 (14.22)	
<10 giga	55.77 (10.66)		62.30 (10.86)		63.15 (15.92)		62.17 (11.19)	
>10 giga, <Unlimited	57.82 (11.63)		61.61 (12.32)		60.14 (16.08)		63.49 (11.96)	
Unlimited	56.78 (10.24)		63.07 (11.34)		63.12 (15.96)		64.66 (11.49)	
**Health Literacy**								
FHL		0.000 ^3^ (15.497)		0.140 ^3^ (3.929)		0.018 ^3^ (8.085)		0.214 ^3^ (3.087)
Limited	58.49 (11.05)		63.11 (11.99)		63.73 (16.36)		64.63 (12.21)	
Marginal	55.69 (10.68)		62.36 (10.75)		61.79 (16.47)		63.53 (11.67)	
Average	56.01 (11.16)		61.21 (12.56)		59.12 (18.33)		63.27 (13.42)	
CHL		0.000 ^3^ (21.367)		0.000 ^3^ (25.717)		0.000 ^3^ (15.947)		0.000 ^3^ (43.464)
Inadequate	50.64 (12.18)		56.96 (12.34)		55.36 (19.41)		55.28 (15.12)	
Problematic	55.82 (11.04)		60.24 (11.80)		59.43 (16.67)		61.21 (11.74)	
Sufficient	57.93 (10.71)		63.79 (11.30)		63.77 (16.44)		65.70 (11.72)	
**Health Behaviors**								
Smoking		0.050 ^2^ (−1.962)		0.764 ^2^ (−0.300)		0.734 ^2^ (−0.340)		0.630 ^2^ (−0.482)
No	57.15 (10.97)		62.45 (11.72)		62.23 (16.90)		63.98 (12.20)	
Yes	54.60 (11.39)		63.24 (10.53)		61.55 (15.80)		63.89 (12.79)	
Alcohol		0.829 ^2^ (−0.216)		0.761 ^2^ (−0.304)		0.819 ^2^ (−0.229)		0.898 ^2^ (−0.129)
No	57.04 (11.13)		62.53 (11.70)		62.22 (16.83)		63.98 (12.34)	
Yes	56.54 (9.43)		62.04 (11.11)		61.78 (16.90)		64.01 (10.98)	
Sleep		0.047 ^2^ (−1.987)		0.293 ^2^ (−1.053)		0.476 ^2^ (−0.712)		0.745 ^2^ (−0.325)
Not Enough	56.43 (10.81)		62.23 (11.22)		62.44 (16.68)		63.94 (12.51)	
Enough	57.70 (11.21)		62.82 (12.17)		61.88 (17.03)		64.02 (11.90)	
Physical exercise		0.092 ^2^ (−1.686)		0.131 ^2^ (−1.511)		0.000 ^2^ (−3.886)		0.001 ^2^ (−3.324)
No	56.27 (10.62)		61.74 (11.27)		59.70 (16.11)		62.38 (11.68)	
Yes	57.51 (11.24)		63.03 (11.90)		63.94 (17.12)		65.10 (12.50)	
**Health Outcome**								
BMI		0.386 ^3^ (1.902)		0.000 ^3^ (23.293)		0.039 ^3^ (6.487)		0.327 ^3^ (2.233)
Underweight	57.00 (10.76)		62.85 (10.41)		61.84 (15.14)		63.21 (11.83)	
Normal Weight	57.26 (11.06)		63.51 (11.60)		63.10 (17.29)		64.37 (12.23)	
Obese	56.24 (11.11)		59.09 (12.51)		59.89 (17.09)		63.68 (12.71)	

^1^ Spearman correlation, ^2^ Mann–Whitney U, ^3^ Kruskal–Wallis; statistics: Spearman correlation: r_s_, Mann–Whitney U: Z, Kruskal–Wallis: χ^2^.

**Table 3:** Multivariate GLMs gamma log-link for the four domains of QOLs with CHL in addition to socioeconomic determinants, HBs, and BMI.

Variables	Physical Health QOL		Psychological Health QOL		Social Relationship QOL		Environmental Health QOL	
	Exp β (95% CI)	*p*-Value	Exp β (95% CI)	*p*-Value	Exp β (95% CI)	*p*-Value	Exp β (95% CI)	*p*-Value
**Socioeconomics**								
Gender								
Female	0.996 (0.967–1.025)	0.772	0.995 (0.967–1.023)	0.733	0.978 (0.935–1.022)	0.264	1.014 (0.986–1.043)	0.326
Male	ref.		ref.		ref.		ref.	
Age	1.011 (0.998–1.025)	0.107	1.003 (0.989–1.016)	0.695	1.011 (0.990–1.032)	0.242	1.012 (0.999–1.026)	0.079
Academic Performance	1.062 (1.002–1.126)	0.040	1.080 (1.019–1.144)	0.009	1.045 (0.956–1.142)	0.268	1.064 (1.004–1.127)	0.039
Major								
Non-Education	0.984 (0.960–1.010)	0.226	0.964 (0.940–0.988)	0.003	0.959 (0.922–0.997)	0.021	0.985 (0.961–1.010)	0.229
Education	ref.		ref.		ref.		ref.	
Father’s Education								
Elementary and Below	0.975 (0.927–1.025)	0.333	1.013 (0.964–1.065)	0.565	0.979 (0.907–1.057)	0.550	0.962 (0.916–1.011)	0.106
Junior and Senior High	0.981 (0.952–1.012)	0.219	0.989 (0.960–1.018)	0.436	0.992 (0.947–1.038)	0.695	0.988 (0.959–1.017)	0.380
University and Postgraduate	ref.		ref.		ref.		ref.	
Mother’s Education								
Elementary and Below	0.986 (0.939–1.034)	0.579	0.958 (0.914–1.005)	0.078	1.004 (0.934–1.081)	0.904	0.966 (0.922–1.013)	0.148
Junior and Senior High	1.002 (0.972–1.032)	0.909	0.990 (0.962–1.020)	0.506	1.003 (0.958–1.049)	0.904	0.974 (0.946–1.003)	0.053
University and Postgraduate	ref.		ref.		ref.		ref.	
Allowance								
Low	0.996 (0.957–1.037)	0.841	0.971 (0.933–1.009)	0.151	0.963 (0.907–1.023)	0.226	0.980 (0.943–1.019)	0.309
Medium	0.987 (0.961–1.015)	0.340	0.981 (0.955–1.008)	0.132	0.978 (0.938–1.019)	0.232	0.952 (0.927–0.978)	0.000
High	ref.		ref.		ref.		ref.	
Internet Access								
No Private Internet	1.021 (0.985–1.058)	0.244	1.006 (0.972–1.042)	0.723	0.994 (0.941–1.049)	0.804	1.025 (0.990–1.061)	0.163
<10 giga	0.992 (0.959–1.027)	0.647	0.999 (0.966–1.034)	0.968	1.020 (0.968–1.075)	0.390	0.976 (0.944–1.009)	0.126
>10 giga, <Unlimited	1.019 (0.984–1.055)	0.280	0.982 (0.949–1.016)	0.273	0.958 (0.909–1.011)	0.080	0.985 (0.952–1.019)	0.364
Unlimited	ref.		ref.		ref.		ref.	
**Health Outcome**								
BMI								
Underweight	1.018 (0.979–1.059)	0.361	1.065 (1.024–1.107)	0.001	1.039 (0.979–1.103)	0.165	0.997 (0.959–1.036)	0.869
Normal Weight	1.018 (0.985–1.051)	0.290	1.071 (1.037–1.106)	0.000	1.050 (0.999–1.103)	0.048	1.010 (0.979–1.043)	0.520
Obese	ref.		ref.		ref.		ref.	
**Health Behaviors**								
Smoking								
No	1.052 (0.993–1.113)	0.094	0.992 (0.938–1.049)	0.763	1.038 (0.950–1.132)	0.336	1.008 (0.953–1.066)	0.768
Yes	ref.		ref.		ref.		ref.	
Alcohol								
No	1.001 (0.953–1.051)	0.965	1.005 (0.958–1.054)	0.829	1.003 (0.931–1.079)	0.931	1.001 (0.954–1.049)	0.971
Yes	ref.		ref.		ref.		ref.	
Sleep								
Not Enough	0.987 (0.962–1.012)	0.296	0.998 (0.973–1.023)	0.870	1.016 (0.977–1.056)	0.376	1.001 (0.977–1.026)	0.926
Enough	ref.		ref.		ref.		ref.	
Physical exercise								
No	0.983 (0.958–1.009)	0.199	0.981 (0.956–1.006)	0.120	0.940 (0.903–0.978)	0.001	0.959 (0.935–0.984)	0.001
Yes	ref.		ref.		ref.		ref.	
**Health Literacy**								
CHL								
Inadequate	0.878 (0.828–0.931)	0.000	0.900 (0.850–0.954)	0.001	0.874 (0.800–0.956)	0.008	0.854 (0.807–0.904)	0.000
Problematic	0.965 (0.938–0.994)	0.015	0.950 (0.924–0.977)	0.000	0.934 (0.894–0.976)	0.001	0.937 (0.911–0.963)	0.000
Sufficient	ref.		ref.		ref.		ref.	

**Table 4:** Multivariate GLM gamma log-link for the four domains of QOL with FHL in addition to socioeconomic determinants, HBs, and BMI.

Variables	Physical Health QOL		Psychological Health QOL		Social Relationship QOL		Environmental Health QOL	
	Exp β (95% CI)	p-Value	Exp β (95% CI)	p-Value	Exp β (95% CI)	p-Value	Exp β (95% CI)	p-Value
**Socioeconomics**								
Gender								
Female	1.000 (0.971–1.029)	0.979	0.997 (0.969–1.026)	0.865	0.981 (0.939–1.026)	0.358	1.017 (0.988–1.047)	0.248
Male	ref.		ref.		ref.		ref.	
Age	1.010 (0.996–1.023)	0.183	1.001 (0.988–1.015)	0.840	1.008 (0.987–1.029)	0.386	1.011 (0.997–1.024)	0.137
Academic Performance	1.078 (1.017–1.144)	0.010	1.088 (1.026–1.153)	0.005	1.054 (0.964–1.152)	0.200	1.076 (1.015–1.141)	0.017
Major								
Non-Education	0.985 (0.960–1.011)	0.249	0.962 (0.938–0.986)	0.002	0.956 (0.919–0.994)	0.013	0.983 (0.958–1.008)	0.170
Education	ref.		ref.		ref.		ref.	
Father’s Education								
Elementary and Below	0.950 (0.903–0.999)	0.042	0.994 (0.945–1.045)	0.778	0.951 (0.882–1.027)	0.162	0.938 (0.892–0.986)	0.007
Junior and Senior High	0.974 (0.945–1.004)	0.083	0.980 (0.951–1.010)	0.176	0.978 (0.934–1.024)	0.303	0.978 (0.949–1.007)	0.113
University and Postgraduate	ref.		ref.		ref.		ref.	
Mother’s Education								
Elementary and Below	0.991 (0.945–1.041)	0.736	0.965 (0.920–1.012)	0.128	1.013 (0.941–1.090)	0.712	0.974 (0.929–1.022)	0.266
Junior and Senior High	0.999 (0.969–1.029)	0.928	0.988 (0.959–1.018)	0.414	1.000 (0.956–1.047)	0.986	0.970 (0.942–0.999)	0.030
University and Postgraduate	ref.		ref.		ref.		ref.	
Allowance								
Low	0.983 (0.945–1.023)	0.384	0.962 (0.925–1.001)	0.065	0.951 (0.896–1.011)	0.114	0.970 (0.933–1.009)	0.142
Medium	0.986 (0.960–1.014)	0.307	0.981 (0.955–1.007)	0.126	0.978 (0.938–1.019)	0.232	0.952 (0.927–0.978)	0.000
High	ref.		ref.		ref.		ref.	
Internet Access								
No Private Internet	1.016 (0.980–1.053)	0.373	1.004 (0.969–1.040)	0.814	0.995 (0.942–1.050)	0.839	1.023 (0.987–1.059)	0.225
<10 giga	0.988 (0.955–1.023)	0.474	0.994 (0.961–1.028)	0.690	1.015 (0.963–1.070)	0.517	0.968 (0.935–1.001)	0.042
>10 giga, <Unlimited	1.013 (0.978–1.049)	0.476	0.978 (0.945–1.012)	0.190	0.955 (0.906–1.008)	0.061	0.978 (0.945–1.013)	0.195
Unlimited	ref.		ref.		ref.		ref.	
**Health Outcome**								
BMI								
Underweight	1.018 (0.978–1.059)	0.375	1.065 (1.024–1.108)	0.001	1.038 (0.977–1.102)	0.171	0.998 (0.960–1.038)	0.915
Normal Weight	1.019 (0.986–1.053)	0.267	1.074 (1.040–1.109)	0.000	1.054 (1.002–1.107)	0.032	1.014 (0.982–1.047)	0.399
Obese	ref.		ref.		ref.		ref.	
**Health Behaviors**								
Smoking								
No	1.045 (0.987–1.107)	0.138	0.982 (0.928–1.039)	0.492	1.025 (0.939–1.118)	0.525	0.992 (0.937–1.050)	0.785
Yes	ref.		ref.		ref.		ref.	
Alcohol								
No	1.012 (0.963–1.063)	0.624	1.012 (0.964–1.063)	0.600	1.018 (0.944–1.097)	0.611	1.010 (0.962–1.061)	0.664
Yes	ref.		ref.		ref.		ref.	
Sleep								
Not Enough	0.990 (0.964–1.015)	0.410	0.998 (0.973–1.024)	0.889	1.019 (0.980–1.059)	0.311	1.002 (0.977–1.027)	0.892
Enough	ref.		ref.		ref.		ref.	
Physical exercise								
No	0.990 (0.964–1.016)	0.437	0.986 (0.961–1.012)	0.270	0.948 (0.911–0.986)	0.004	0.964 (0.940–0.990)	0.006
Yes	ref.		ref.		ref.		ref.	
**Health Literacy**								
FHL								
Limited	1.048 (1.010–1.086)	0.011	1.032 (0.996–1.069)	0.094	1.075 (1.017–1.135)	0.009	1.022 (0.986–1.059)	0.249
Marginal	0.994 (0.959–1.031)	0.761	1.017 (0.981–1.054)	0.362	1.039 (0.983–1.098)	0.168	1.001 (0.966–1.038)	0.940
Average	ref.	-	ref.	-	ref.	-	ref.	-

## Data Availability

The corresponding author can provide the data used in this study upon request.
